# Abnormal functional connectivity of the occipital thalamus with the superior occipital gyrus is associated with mild cognitive impairment in elderly individuals with primary insomnia

**DOI:** 10.1002/brb3.3411

**Published:** 2024-02-05

**Authors:** Lin Zhang, Linxin Bai, Zhenxing Guo, Jiahui Gao, Jingsong Wu, Jia Huang, Zhizhen Liu

**Affiliations:** ^1^ College of Rehabilitation Medicine Fujian University of Traditional Chinese Medicine Fuzhou Fujian China; ^2^ National‐Local Joint Engineering Research Center of Rehabilitation Medicine Technology Fujian University of Traditional Chinese Medicine Fuzhou Fujian China

**Keywords:** mild cognitive impairment, primary insomnia, rs‐fMRI, thalamus

## Abstract

**Study objectives:**

Primary insomnia (PI) triggers a decline in cognitive function, and the thalamus plays an integral role in this process; however, the mechanisms are unclear. The purpose of this study was to investigate the altered functional connectivity (FC) of the thalamus in PI patients with mild cognitive impairment (MCI) and to explore the potential neural mechanisms of thalamic involvement in these patients.

**Methods:**

This case–control study was conducted in older adults from various communities in Fuzhou, China with a PI diagnosis. These participants underwent neuropsychological assessment and were matched in a 1:2 ratio to the healthy control (HC) group and the PI group according to sex, age, and education level. Resting‐state functional magnetic resonance imaging was used to explore changes in thalamic FC in PI patients. To further compare changes in thalamic and whole‐brain FC, we further divided the PI group into cognitively normal patients and patients with MCI according to the diagnostic criteria for MCI. The relationship between abnormal FC and cognitive function was investigated.

**Results:**

The 28 HCs and 58 participants with PI showed significant differences in Montreal Cognitive Assessment (MoCA) scores (*p* = .044). In comparison to the HC group, the PI group showed enhanced FC of the occipital thalamus with the left inferior occipital gyrus, right lingual gyrus, left middle temporal gyrus, left superior marginal gyrus, left dorsolateral superior frontal gyrus, and right anterior central gyrus. The MoCA total score and the executive function, attention, and abstraction scores of PI patients with MCI (PI–MCI) were worse than those of PI patients without MCI. In comparison to the simple PI group, FC was enhanced in the PI–MCI group between the left occipital thalamus and the middle occipital gyrus, and between the right occipital thalamus and the right superior frontal gyrus, left middle frontal gyrus, right superior occipital gyrus, and orbital inferior frontal gyrus. There was a significant negative correlation between the MoCA total score and the enhanced FC between the right occipital thalamus and right superior occipital gyrus (*r* = −.419, *p* = .042).

**Conclusion:**

The early onset of cognitive impairment in patients with PI is associated with altered FC between the thalamus and the cortex. Enhanced FC between the thalamus and the visual cortex, that is, the superior occipital gyrus, which is involved in attentional processing, may play a role in the early onset of cognitive impairment in insomnia patients. Moreover, due to the fact that PI patients “overdraw” the compensatory capacity of these brain regions earlier, the PI–MCI may fall into the abyss of “decompensation” faster and face more severe cognitive impairments.

## Graphical abstract

Neuropsychological assessment and resting‐state fMRI were conducted on healthy elderly individuals and primary insomnia patients. The results revealed increased functional connectivity between the right thalamus and the superior occipital gyrus in primary insomnia patients with MCI. This abnormal alteration is not only associated with early cognitive impairment in primary insomnia patients but may also accelerate the brain's decompensation mode.

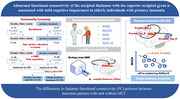

## 1 LIMITATIONS

There are several limitations of this study. First, we used a cross‐sectional group comparison design, and the causality of the comorbidity relationship can therefore not be assessed. Second, because our sample size was relatively small, the results of this study need to be further validated in a study with a larger sample. Third, in this study, we measured cognitive function with only the MoCA, and more tests of different cognitive domains are needed in the future to further explore the effects of insomnia on different aspects of cognitive function. Additionally, further longitudinal imaging studies are needed to explore the changes in functional activity of the thalamus before and after interventions, which can also help to provide new ideas for the clinical treatment of insomnia patients with MCI. Finally, in this study, the number of male participants was only eight in some groups (HC, PI–MCI). Therefore, male participants were under‐represented, making further statistical analysis of sex differences difficult. As sex differences may play a role in the comorbidity between insomnia and cognitive, further research, including more male participants, should be considered.

## 2 INTRODUCTION

Primary insomnia (PI) is one of the most common sleep disorders in the elderly population (Kamel & Gammack, 2006). It is characterized by difficulties in initiating and maintaining sleep, and it causes a range of cognitive dysfunction and decreased cognitive control (Baril et al., 2022; Zhang et al., 2023), mainly involving attention, memory, and executive functions (Brownlow et al., 2020). Studies have found that sleep disorders can increase the risk of cognitive impairment by increasing brain levels of Aβ and tau proteins and reducing metabolite clearance (Boespflug et al, 2018). A meta‐analysis based on global clinical data pooling 23 cohort studies, including 260,915 participants, showed that insomnia increased the risk of cognitive impairment by 27% (Xu et al., 2020). Thus, insomnia is likely to damage the brain and affect cognitive function in a long‐term and severe manner.

It has been found that the initiation and maintenance of sleep is regulated by a delicate balance of inhibition and activation in the thalamus (Lugaresi et al., 1986). PI is thought to be the result of an overall increase in cortical and physiological arousal during the sleep‐wake cycle, and substantial evidence confirms that the thalamus plays an integral role in arousal (Lugaresi, 1992; Min, 2010). The presence of structural and metabolic changes in the thalamus or brain circuits involving the thalamus in patients with insomnia or sleep deprivation has been validated in neuroimaging studies (Koo et al., 2017; Liu et al., 2014; Nofzinger et al., 2004). The thalamus, in turn, is critical for cognitive function, contributing in particular to cortical functions and higher cognitive functions from learning and memory to flexible adaptation (Wolff & Vann, 2019). Studies have shown that sleep deprivation drastically reduces the activity of the corticothalamic network that mediates attention and higher order cognitive processes (Thomas et al., 2000) and that thalamic lesions can directly or indirectly affect the hippocampus, which in turn impairs memory (Aggleton et al., 2010). This suggests that the thalamus may be a key brain region in insomnia‐induced cognitive impairment.

Mild cognitive impairment (MCI), a prodromal state of dementia, represents a window of opportunity to combat further cognitive impairment. Abnormal thalamic functional alterations in patients with insomnia have been identified in previous neuroimaging studies (Lee et al., 2018; Li et al., 2019; Yan et al., 2018); however, the differences in thalamic functional connectivity (FC) patterns between insomnia patients with and without MCI are unclear. Some scholars have investigated abnormal FC alterations in patients with both PI and MCI (Luo et al., 2022; Pang et al., 2017) and found that they showed extensive FC enhancement manifestations in memory‐related regions. Another study of objective sleep monitoring in older adults with probable MCI found that higher sleep fragmentation was associated with enhanced FC in the somatosensory network, the frontoparietal network, which is involved in attentional and somatosensory processes (Hsu et al., 2022). However, the importance of the thalamus has been mostly ignored, and exploratory analyses have only been performed on a large scale (the whole brain) in patients with both PI and MCI. Therefore, further studies are needed to explore the potential role of the thalamus in early cognitive impairment in patients with insomnia.

Based on the exploratory neuroimaging results in insomnia patients in existing studies, we hypothesized that PI patients with MCI (PI–MCI) would enhance in thalamic FC patterns, and that this abnormal alteration would be closely related to the severity of cognitive decline. The aim of this study was to deeply explore the thalamic FC characteristics of PI patients with MCI and to explore the potential pathogenic involvement of the thalamus in MCI in insomnia patients, which will help us to better understand the role of cognitive deficits in the progression of insomnia.

## 3 METHODS

### 3.1 Participants

Elderly participants were recruited to undergo sleep quality assessment and cognitive screening from June 2019 to December 2020 from various communities in Fuzhou City, Fujian Province, China. After application of the inclusion and exclusion criteria, eligible participants underwent MRI at the Rehabilitation Hospital affiliated with Fujian University of Traditional Chinese Medicine. The study was approved by the Medical Ethics Committee of the Rehabilitation Hospital Affiliated to Fujian University of Traditional Chinese Medicine (2019KF‐002‐02), and all participants signed an informed consent form before the start of the study procedures.

Healthy older adults with normal sleep and patients with PI were included. Diagnosis of PI involved the following criteria: (1) diagnosis of PI according to the Diagnostic and Statistical Manual of Mental Disorders, fifth edition criteria (Riemann et al., 2022); (2) Pittsburgh sleep quality index (PSQI) scores ≥6; and (3) no history of significant cognitive disorders or other psychiatric and neurological disorders. The inclusion criteria were as follows: (1) aged 60–75 years; (2) normal ability to perform basic self‐care and activities of daily living; (3) ability to understand and cooperate with the study, voluntarily participate, and sign of an informed consent form; (4) no significant behavioral and language disorders; and (5) right‐handed.

The exclusion criteria were as follows: (1) the presence of dementia; (2) the history of apnea syndrome disease; (3) a score >8 on the Geriatric Depression Scale–short form GDS‐15 or a history of depression; (4) previous traumatic brain injury, brain tumor, cerebral infarction, cerebral hemorrhage, intracranial infection, Parkinson's disease, epilepsy, or other neurological disorders; (5) previous anxiety, depression, schizophrenia, or other psychiatric disorders; (6) the use of neuroleptic medication, antidepressants, tranquilizers, or antipsychotics in the last two weeks; or (7) refusal to participate in the current study or poor cooperation with the study.

Healthy older adults and PI patients were assigned to groups in a 1:2 ratio and matched in terms of age, sex, and education level. A total of 30 healthy elderly individuals (healthy control [HC] group) and 60 PI patients (PI group) were finally included. Based on the presence or absence of MCI, we further divided PI–MCI and those without MCI (simple‐PI). The diagnostic criteria for MCI were based on the MCI diagnostic criteria in the 2018 Chinese guidelines for the diagnosis and treatment of dementia and cognitive impairment (Chinese Dementia and Cognitive Disorders Diagnosis and Treatment Guidelines Writing Group, 2018), which are as follows: (1) the complaint of substantial memory loss or memory impairment; (2) objective evidence of cognitive impairment in cognitive testing, as shown by a Montreal Cognitive Assessment (MoCA) score ≤24 points (or ≤19 points if the number of years of education is ≤6); (3) normal or slightly impaired ability to perform activities of daily living but maintains the ability to independently perform activities of daily living (Lawton instrumental activities of daily living [IADL] scale score > “norm–2 standard deviations”); and (4) the absence of dementia.

**FIGURE 1:**
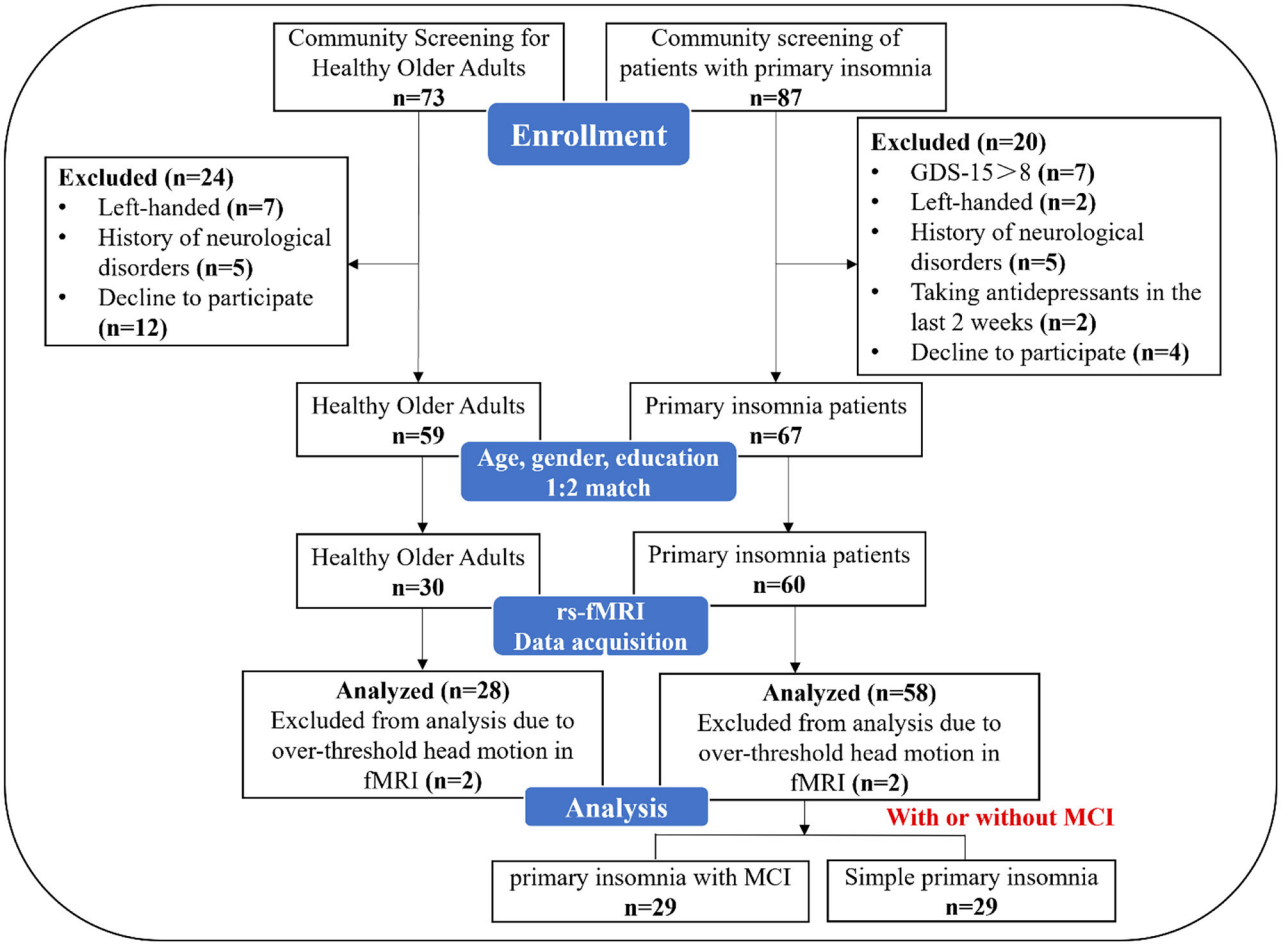
Flowchart of participant recruitment and group allocation.

Participant recruitment and group allocation are shown in Figure 1.

### 3.2 Assessment of clinical characteristics

The clinical characteristics we focused on included subjective sleep quality, the neuropsychological profile, and cognitive function. All participants completed a battery of tests prior to undergoing MRI, including the following:

(1) Pittsburgh sleep quality index scale (PSQI)

The participants assessed their sleep quality in the last month using the internationally recognized PSQI (Mollayeva et al., 2016). The Chinese version of the PSQI has been tested for reliability and validity and is suitable for assessing sleep quality in the Chinese population (Lu et al., 2014). The PSQI consists of 19 self‐report items that comprise 7 dimensions, namely, sleep quality, sleep latency, sleep duration, sleep efficiency, sleep disorders, hypnotic drug use, and daytime dysfunction. Each item is scored on a scale from 0 to 3, and the PSQI total score is calculated as the sum of scores on all dimensions, with higher scores indicating worse quality of sleep. A score of 0–5 indicates good sleep quality; scores ≥6 indicate a sleep disorder.

(2) Montreal cognitive assessment (MoCA, Fuzhou version)

The Fuzhou version of the MoCA was used to assess the overall cognitive function of the subjects in a face‐to‐face context. A preliminary analysis demonstrated that the Fuzhou version of the MoCA has good reliability and structural validity with satisfactory factor loadings on the corresponding factors (Gagnon et al., 2010), including eight cognitive domains: executive function, visuospatial skills, memory, attention, verbal fluency, abstraction, calculation, and orientation.

(3) Ascertain dementia eight‐item questionnaire (AD8)

The AD8 was developed by the University of Washington in 2005 and contains eight items (Denny et al., 2021). The Chinese version of the scale uses a score ≥2 as the cutoff value for cognitive impairment, with a sensitivity of 85.7% and a specificity of 77.6% (Wang et al., 2020). Because the Chinese version of the AD8 is less time‐consuming and is easy for older adults to understand and complete self‐assessments, it has good potential for widespread use in community and nonspecialized medical settings such as primary care settings.

(4) Instrumental activities of daily living (IADL) scale

Developed by Lawton and Brody (1969), this scale exhibits good reliability and validity. The scale contains eight items: telephone use, shopping, food preparation, household maintenance, laundry, transportation, medication management, and financial management. The total score ranges from 0 to 23, with higher scores representing better ability to perform instrumental activities of daily living. A score more than 2 standard deviations below the norm indicates that the ability to perform activities of daily living is severely impaired.

(5) Geriatric depression scale‐15 (GDS‐15)

Simplified from the scale developed by Burke et al. (1991), the Chinese version of the GDS‐15 has an internal consistency (Cronbach's *α* coefficient) of .82 (Mei, 1999). The scale contains 15 items, of which Items 1, 5, 7, 11, and 13 are reverse scored. Items are given a score of 0 or 1, with the total score ranging from 0 to 15. The higher the score, the more severe the depressive symptoms. A GDS‐15 score >8 indicates the presence of depressive symptoms.

### 3.3 MRI acquisition and processing

Brain MRI data were obtained using a Siemens Prisma 3.0 Tesla system at the Affiliated Rehabilitation Hospital of Fujian University of Traditional Chinese Medicine. Subjects were asked to stay awake and remain motionless with eyes closed during the scan. A shorter rs‐fMRI scan with 240 data points was performed on the subjects, and each scan with 240 acquisitions took 8 min. Resting‐state functional MR images were acquired with the following parameters: TR = 2000 ms, TE = 30 ms, flip angle = 90°, slice thickness = 3.5 mm, matrix = 64 × 64, voxel size = 3.6 × 3.6 × 3.9 mm^3^, field of view (FOV) = 224 × 224 mm^2^, and number of slices = 37.

The following parameters were used to collect the T1‐weighted sequence: TR = 2530 ms, TE = 2.51 ms, flip angle = 7°, slice thickness = 1.0 mm, matrix size = 256 × 256, slice number = 192 contiguous slices, voxel size = 1 × 1 × 1 mm^3^, and FOV = 256 × 256 mm^2^.

The rs‐fMRI data were preprocessed using SPM12 and Data Processing & Analysis of Brain Imaging toolbox (http://www.restfmri.net/) (Yan et al., 2016) with the following procedures: (1) removal of the first 10 time points and (2) slice timing and head motion correction according to the realignment curve. Data from participants with head motion greater than 3 mm in translation or greater than 3° in rotation were excluded. (3) Images were registered to the Montreal Neurological Institute (MNI) space via the normalization of the functional time‐series MNI EPI template to account for individual anatomical differences; (4) a linear regression was used to remove linear drift, white matter signals, CSF signals, and head movement (Friston 24 parameter model) were regressed out as covariables to remove physiological influences; (5) smoothing was conducted with 6 mm full‐width at half maxima the Gaussian kernel; and (6) a bandpass filter of 0.01–0.1 Hz was applied.

### 3.4 Functional connectivity analysis

The regions of interest (ROIs), the left and right thalamus, were chosen because they have shown abnormal activity in PI patients, as demonstrated by previous studies. These ROIs were identified based on the automated anatomical labeling atlas using the software REST (Wang et al., 2017). Furthermore, based on the Brainnetome atlas (Fan et al., 2016), the thalamus was divided into eight subregions, including the medial prefrontal thalamus (mPFtha), premotor thalamus (mPMtha), sensory thalamus (Stha), temporal thalamus (rTtha), posterior parietal thalamus (PPtha), occipital thalamus (Otha), caudotemporal thalamus (cTtha), and lateral prefrontal thalamus (IPFtha).

Each of the left and right thalamus subregions was defined as seed ROIs. These brain areas were then employed to perform a voxel‐wise whole‐brain FC analysis to further highlight key FC. First, we resampled the seed regions’ masks into a voxel size of 3 × 3 × 3 mm^3^ and extracted the mean time series. Second, we applied Pearson's correlation coefficient to estimate FC between the seed region's averaged time series and remaining brain voxels. Then Fisher's *z* transformation was performed to convert FC to *z* value to improve normality. Finally, we obtained *z*‐FC maps of each subject for group statistics.

### 3.5 Data analysis

Statistical analyses were conducted with IBM SPSS version 26.0 for group assignment. Continuous data (demographic characteristics and baseline outcome measures) were examined using *t*‐tests or Mann–Whitney *U* tests according to the normality of data distribution. Categorial data were compared between groups using chi‐square tests.

The two‐sample *t*‐test was also adopted to identify significant between‐group differences in FC using zFC maps of the thalamus across the two groups’ ROIs. A threshold of voxel‐wise *p* < .005 (uncorrected) and cluster‐wise *p* < .05 (false discovery rate corrected) were used for the analyses. Then, partial correlation analysis was performed to clarify the relationships between neuropsychological outcomes and the abnormal FC of significantly different regions. Age, sex, education level, AD8 scores, and GDS‐15 scores were included in the analysis as covariates.

## 4 RESULT

### 4.1 Characteristics of participants

A total of 90 subjects participated in neuroimaging scans, of whom 4 were excluded because of poor imaging quality due to head movements (translations greater than 3 mm in any axis and angular rotations greater than 3° in any axis) that resulted in poor imaging quality. Eighty‐six subjects were finally included, including 28 HCs and 58 PI patients.

As shown in Table 1, the two groups did not significantly differ (*p* > .05) in terms of sex, age, education level, or IADL scores but did significantly differ (*p* < .05) in terms of AD8 score, GDS‐15 score, PSQI total score, and MoCA total score.

**TABLE 1:** Demographic and clinical characteristics of primary insomnia (PI) patients and healthy controls.

Characteristic	HC (*n* = 28)	PI	*t*/*Z*/*χ* ^2^	*p*
		(*n* = 58)		
Sex, *n* (%)^a^			.49	.483
Male	8 (28.60)	21 (36.20)		
Female	20 (71.40)	37 (63.80)		
Age (years)^b^	64.61 ± 5.34	64.79 ± 5.47	−1.49	.882
Education (years)^c^	12 (8.25–14)	9.5 (8–12)	−1.23	.219
IADL score^c^	23 (23–23)	23 (22–23)	−1.14	.253
AD8 score^c^	0 (0–1)	1 (0.75–3)	3.59	**<.001**
GDS‐15 score^c^	1 (1–2)	3 (2–4.25)	3.24	**.001**
PSQI score^c^	4 (3–5)	8 (6–11)	7.53	**<.001**
MoCA score^c^	24.5 (21–26)	22 (20–24)	−2.01	**.044**
Visuospatial skills^c^	4 (3–5)	4 (3–5)	−1.39	.164
Naming^c^	3 (3–3)	3 (2–3)	−1.60	.110
Attention^c^	6 (5–6)	6 (5–6)	−.04	.965
Language^c^	2 (1.25–3)	2 (1–2)	−1.39	.166
Abstraction^c^	1 (1–2)	1 (0–2)	−1.61	.106
Delayed recall^c^	2 (2–3)	2 (1–3)	−.55	.583
Orientation^c^	6 (6–6)	6 (5.75–6)	−1.46	.145

*Note*: Bold text indicates a *p* value of <.05.

Abbreviations: HC, healthy controls; IADL, instrumental activities of daily living; AD8, ascertain dementia eight‐item questionnaire; GDS‐15, 15‐item geriatric depression; PSQI, Pittsburgh sleep quality index; MoCA, Montreal Cognitive Assessment.

^a^ Using the chi‐square.

^b^ Using *t*‐test revealed, plus–minus values are mean ± standard deviation.

^c^ Using Mann–Whitney *U* test because the data did not conform to a normal distribution. Median values (25th–75th percentiles) instead of mean values were used to describe results.

### 4.2 Alterations in FC in PI patients

**FIGURE 2:**
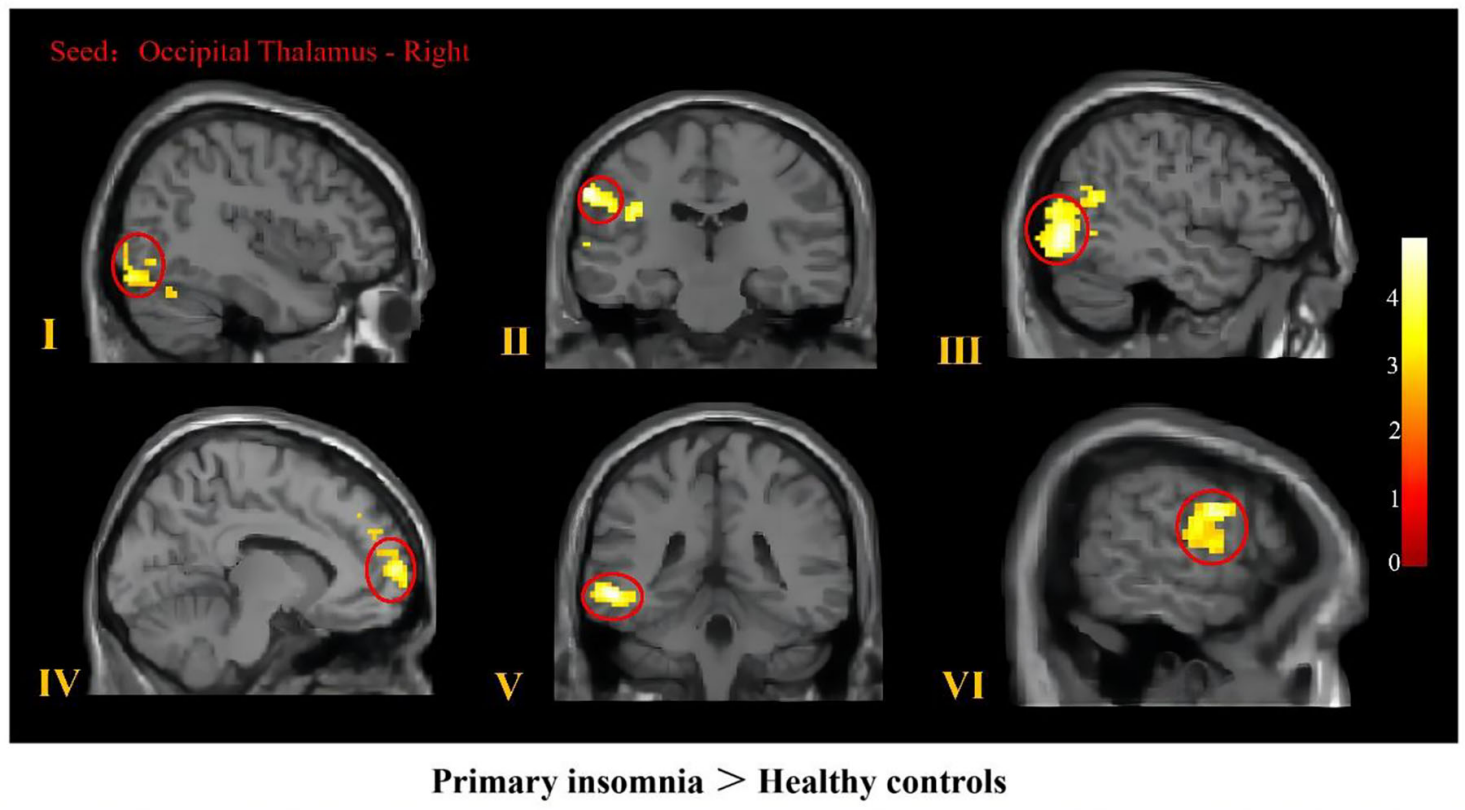
Regions exhibiting abnormal functional connectivity (FC) with the occipital thalamus in primary insomnia (PI) patients. Resting‐state FC results using the right occipital thalamus as the seed. The PI group showed increased FC in the right lingual gyrus(I), left superior marginal gyrus(II), left inferior occipital gyrus(III), left dorsolateral superior frontal gyrus(IV), left middle temporal gyrus(V), and right anterior central gyrus(VI) with the right occipital thalamus compared with the HC group. The statistical threshold was a voxel‐wise uncorrected *p* < .001, with a cluster‐wise false discovery rate corrected *p* < .05. The color bar reflects *t*‐values.

The resting‐state FC results are shown in Table 2 and Figure 2. We found that, compared with the HC group, the PI group exhibited enhanced FC of the right occipital thalamus with the left inferior occipital gyrus, right lingual gyrus, left middle temporal gyrus, left superior marginal gyrus, left dorsolateral superior frontal gyrus, and right anterior central gyrus.

**TABLE 2:** Brain regions showing abnormal functional connectivity (FC) with the occipital thalamus in primary insomnia (PI) patients.

Contrast	Brain area	MNI coordinates (mm)			Peak‐level *t* value	*p* Value	Cluster size
		*x*	*y*	*z*			
Seed: Left occipital thalamus							
None							
Seed: Right occipital thalamus							
PI > HC	Occipital_Inf_L	−48	−75	−3	4.36	<.001	563
PI > HC	Lingual_R	42	−84	−18	3.86	.019	120
PI > HC	Temporal_Mid_L	−57	−39	−9	4.74	.003	187
PI > HC	SupraMarginal_L	−60	−21	36	4.39	<.001	320
PI > HC	Frontal_Sup_Medial_L	−9	63	9	4.14	.019	123
PI > HC	Postcentral_R	63	−3	24	4.39	.003	186

*Note*: Voxel‐wise *p* value <. 005, FDR‐corrected cluster‐wise *p* value <.05.

Abbreviations: HC, healthy controls; Inf, inferior; L, left; Mid, middle; MNI, Montreal Neurological Institute; R, right; Sup, superior.

There was no significant group difference in the FC at the seed sites of the left and right thalamus, the medial prefrontal thalamus (mPFtha), premotor thalamus (mPMtha), sensory thalamus (Stha), temporal thalamus (rTtha), posterior parietal thalamus (PPtha), caudotemporal thalamus (cTtha), and lateral prefrontal thalamus (IPFtha).

### 4.3 Characteristics of PI–MCI subjects

To investigate the altered thalamic FC in PI patients with MCI, further subgroup analysis was conducted. There were no significant differences in sex, age, IADL scores, GDS‐15 scores, or PSQI scores between the two groups (*p* < .05). However, the groups significantly differed in years of education, AD8 scores, MoCA total scores, and scores on the MoCA subdomains of visuospatial skills, attention, and abstraction (*p* < .05), as shown in Table 3.

**TABLE 3:** Demographic and clinical characteristics of primary insomnia patients with mild cognitive impairment (PI–MCI) and simple‐PI patients.

Variable	Simple‐PI (*n* = 29)	PI–MCI	*t*/*Z*/*χ* ^2^	*p* Value
		(*n* = 29)		
Sex, *n* (%)^a^			1.87	.172
Male	13 (44.80)	8 (27.60)		
Female	16 (55.20)	21 (72.40)		
Age (years)^b^	64.24 ± 4.98	65.34 ± 5.95	−1.49	.882
Education (years)^c^	12 (9–12)	9 (8–11.5)	−2.45	**.014**
IADL score^c^	23 (22–23)	23 (22.5–23)	.96	.339
AD8 score^c^	1 (0–1)	2 (2–4)	4.89	**<.001**
GDS‐15 score^c^	2 (1–3.5)	4 (2–5.5)	1.71	.088
PSQI score^c^	8 (6.5–10)	8 (6–12.5)	.30	.764
MoCA total score^c^	23 (21–27)	21 (19.5–23)	−3.07	**.002**
Visuospatial skills^c^	4 (3.5–5)	3 (2.5–4)	−2.87	**.004**
Naming^c^	3 (3–3)	3 (2–3)	−1.36	.174
Attention^c^	6 (6–6)	5 (5–6)	−2.53	**.011**
Language^c^	2 (1–2.5)	2 (1–2)	−.76	.450
Abstraction^c^	1 (1–2)	1 (0–1)	−2.70	**.007**
Delayed recall^c^	2 (2–4)	2 (1–2.5)	−1.91	.056
Orientation^c^	6 (6–6)	6 (5–6)	−.61	.543

*Note*: Bold text indicates a *p*‐value of <.05.

Abbreviations: AD8, ascertain dementia eight‐item questionnaire; GDS‐15, 15‐item geriatric depression; IADL, instrumental activities of daily living; MoCA, Montreal Cognitive Assessment; PSQI, Pittsburgh sleep quality index; simple‐PI, simple primary insomnia patients.

^a^ Using the chi‐square.

^b^ Using *t*‐test revealed, plus–minus values are mean ± standard deviation.

^c^ Using Mann–Whitney *U* test because the data did not conform to a normal distribution. Median values (25th–75th percentiles) instead of mean values were used to describe results.

### 4.4 Differences in FC between PI–MCI and simple‐PI patients

**FIGURE 3:**
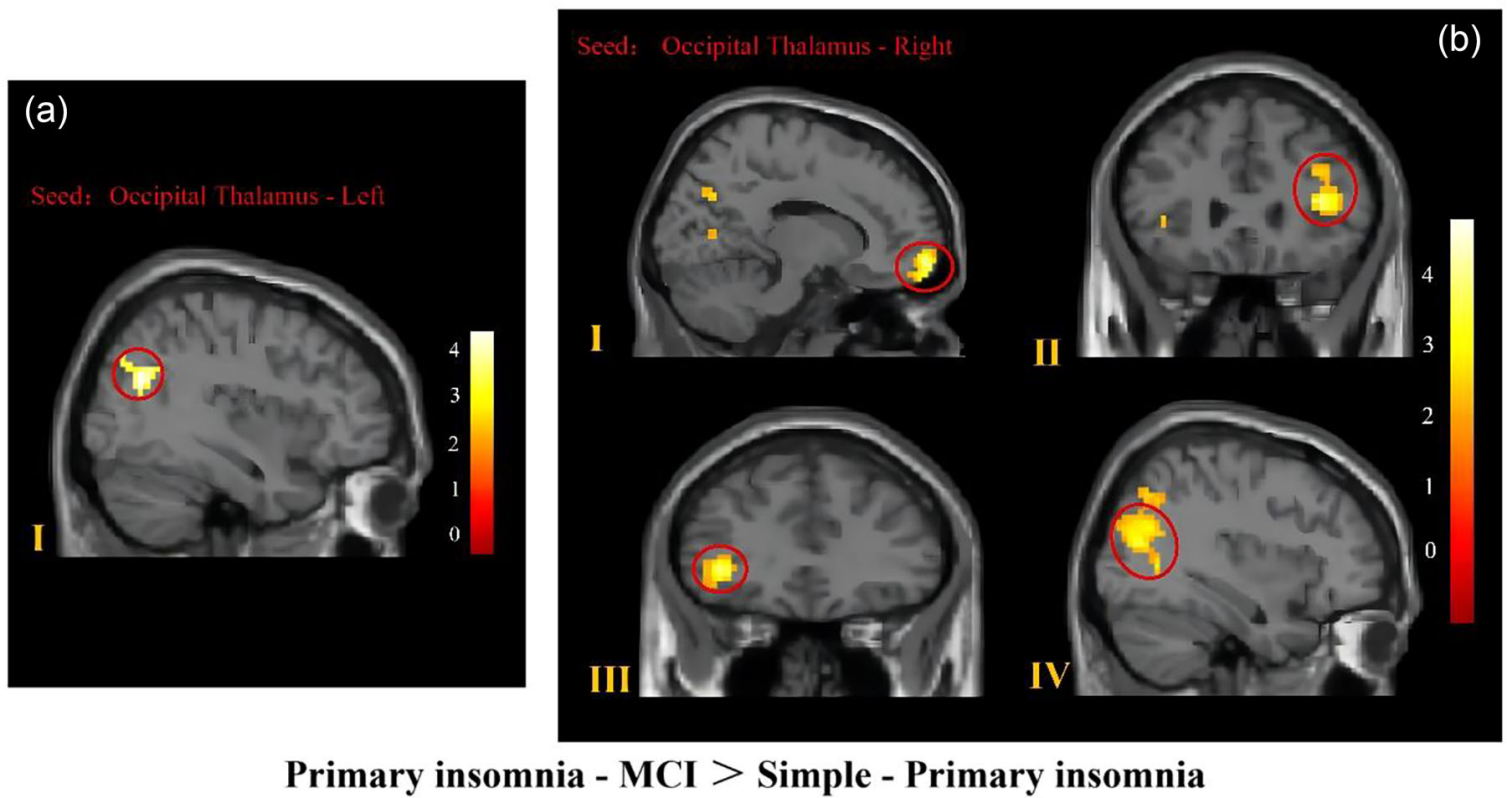
Regions with different functional connectivity (FC) with the occipital thalamus between primary insomnia patients with mild cognitive impairment (PI–MCI) and simple PI (SPI) patients. (a) Resting state FC results using the left occipital thalamus as the seed. The PI–MCI group showed increased FC in the right middle occipital gyrus (A‐I) with the left occipital thalamus compared with the SPI group. (b) Resting state FC results using the right occipital thalamus as the seed. The PI–MCI group showed increased FC in the right superior frontal gyrus (b‐I), left middle frontal gyrus (b‐II), inferior orbital frontal gyrus (b‐III), and right superior occipital gyrus (b‐IV) with the right occipital thalamus compared with the SPI group. The statistical threshold was a voxel‐wise uncorrected *p* < .001, with a cluster‐wise false discovery rate corrected *p* < .05. The color bar reflects *t*‐values.

We found that the FC between the left occipital thalamus and the right middle occipital gyrus as well as that of the right occipital thalamus with the right superior frontal gyrus, left middle frontal gyrus, right superior occipital gyrus, and inferior orbital frontal gyrus was enhanced in the PI–MCI group compared to the simple‐PI group, as shown in Table 4 and Figure 3.

**TABLE 4:** Brain regions showing different functional connectivity (FC) with the occipital thalamus between the primary insomnia patients with mild cognitive impairment (PI–MCI) and the simple‐PI groups.

Contrast	Brain area	MNI coordinates (mm)			Peak‐level *t* value	*p* Value	Cluster size
		*x*	*y*	*z*			
Seed: Left occipital thalamus							
PI–MCI > SPI	Occipital_Mid_R	39	−66	24	3.85	.046	121
Seed: Right occipital thalamus							
PI–MCI > SPI	Frontal_Sup_R	45	51	−15	5.18	<.001	266
PI–MCI > SPI	Frontal_Mid_L	−36	57	−15	4.62	.002	192
PI–MCI > SPI	Occipital_Sup_R	27	−81	45	5.39	<.001	1172
PI–MCI > SPI	Frontal_Inf_Tri_R	39	27	12	4.63	.004	167

*Note*: Voxel‐wise *p* value <. 005, FDR‐corrected cluster‐wise *p* value <.05.

Abbreviations: Inf, inferior; L, left; Mid, middle; MNI, Montreal Neurological Institute; R, right; SPI, simple primary insomnia; Sup, superior; Tri, triangular.

There was no significant group difference in the FC at the seed sites of the left and right thalamus, the medial prefrontal thalamus (mPFtha), premotor thalamus (mPMtha), sensory thalamus (Stha), temporal thalamus (rTtha), posterior parietal thalamus (PPtha), caudotemporal thalamus (cTtha), and lateral prefrontal thalamus (IPFtha).

### 4.5 Correlation between alterations in FC and clinical scale scores

**FIGURE 4:**
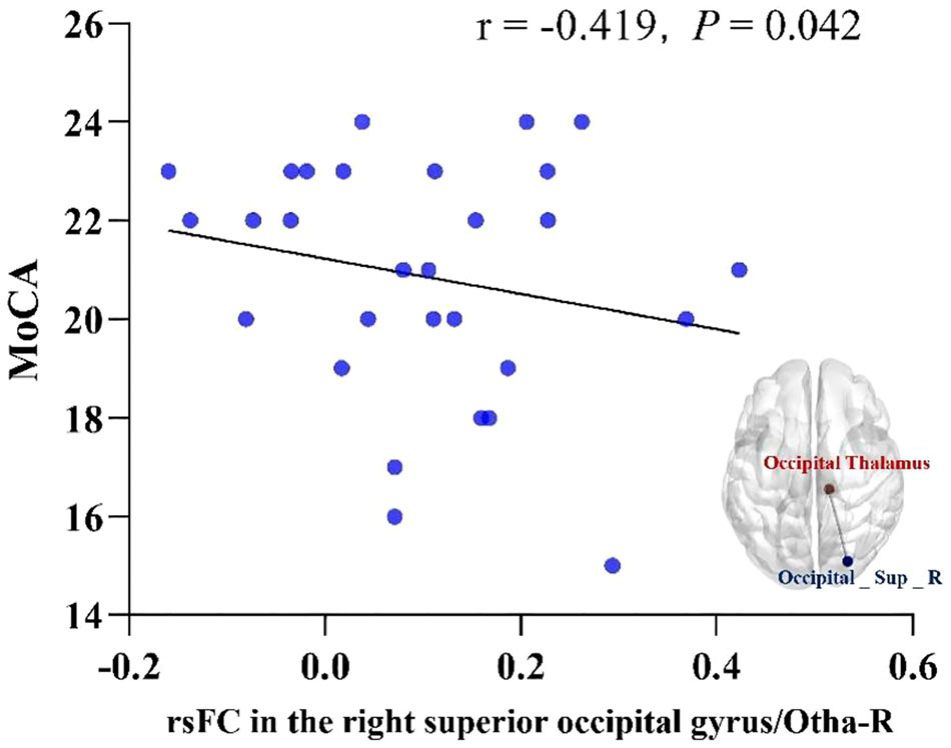
Correlation of functional connectivity (FC) between the right occipital thalamus and right superior occipital gyrus with Montreal Cognitive Assessment (MoCA) total scores. Scatter plots showing a significant negative correlation between the MoCA total score and FC that between the right occipital thalamus and right superior occipital gyrus (*r* = −.419, *p* = .042) adjusted for age, gender, education, Geriatric Depression Scale (GDS‐15), and ascertain dementia eight‐item questionnaire (AD8). Otha‐R, right occipital thalamus.

Brain regions significantly differing in FC were included in partial correlation analyzes with cognitive function assessment results that significantly differed among groups, controlling for age, sex, education level, GDS‐15 scores, and AD8 scores. The results showed a significant negative correlation between the MoCA total score and FC between the right occipital thalamus and right superior occipital gyrus (*r* = −.419, *p* = .042) (Figure 4).

## 5 DISCUSSION

In this study, we investigated altered thalamic FC in PI–MCI. The results of the study supported our hypothesis: Compared to simple‐PI patients, PI–MCI patients showed a specific reorganization of thalamic FC patterns (specifically, a significant enhancement of FC between the right occipital thalamus and the right superior occipital gyrus), and this abnormal FC was negatively correlated with the severity of cognitive decline. This may indicate that the thalamocortical loop is involved in the cognitive regulation of PI patients, in which the enhanced FC between the thalamus and the superior occipital gyrus, which is involved in attentional processing, may play a role in the pathological process of cognitive impairment that occurs early in PI patients.

### 5.1 Altered occipital thalamic FC in PI patients

The present study confirmed that thalamic FC is altered in PI patients compared to HCs and that thalamocortical FC is significantly increased. This is the same as the findings of Lee et al. (2018). The thalamus plays a crucial role in the sleep–wake system. The thalamus and cortex are closely connected by neuronal fibers radiating from the thalamus to the cortex (Sherman, 2016). A significant increase in FC of the bilateral thalamus was observed in healthy subjects after sleep deprivation and in insomnia patients (Li et al., 2017; Zhu et al., 2016). A recent review showed (Fasiello et al., 2022) that insomnia symptoms are associated with impaired intra‐ and interhemispheric connectivity of brain regions associated with hyperarousal, cognitive function, or other factors. This suggests that the increased thalamic FC may reflect hyperarousal in insomnia patients. Indeed, previous physiological, neuroimaging, and neurocognitive studies have confirmed that chronic insomnia patients can present with hypervigilance and/or hyperarousal (Harvey, 2002; Nofzinger et al., 2004). Hyperarousal is defined as excessive cortical, somatic, and cognitive activation resulting in increased sensory information processing and an inability to initiate or maintain sleep (O'byrne et al., 2014; Perlis et al., 2001). These studies suggest that the excessive arousal model may reflect the central precipitating or perpetuating factor in insomnia. Our findings revealed enhanced occipital thalamic FC in insomnia patients, which may provide some evidence for the excessive arousal theory.

### 5.2 Altered occipital thalamic FC in PI–MCI patients

We observed a significant enhancement of FC between the occipital thalamus and the right superior occipital gyrus in PI–MCI patients compared to simple‐PI patients. The superior occipital gyrus is associated with the processing of visual information and is capable of analyzing and synthesizing visual stimuli. It is well known that the occipital lobe, a visual processing center, is mainly involved in complex visual perception processes (Saionz et al., 2020); recent studies have shown (Fernández & Carrasco, 2020) that the visual areas of the occipital cortex are subordinated to the attention network, which prioritizes the content of received information and processes images selectively. This suggests that visual information processing in the occipital cortex plays a key functional role in selective attention. Similarly, some scholars have found (Xia et al., 2014) that patients with attention‐deficit/hyperactivity disorder have significantly lower local and regional efficiency in frontal and occipital regions during visual information processing, and the activation of the inferior frontal gyrus, orbitofrontal cortex, and occipital cortex was significantly reduced during cognitive processing (Schulz et al., 2017).

Selective attention is an important component of human cognitive ability. MCI patients often exhibit a deficit in visual attention that is intermediate between the performance of healthy older adults and adults with Alzheimer's disease (AD). A study exploring visual attention in MCI patients found (Okonkwo et al., 2008) that worse visual attention was associated with worse overall cognitive function. This is similar to our findings, whereas stronger FC may reflect more additional attentional processing. Impairments in visual attention and visual information processing have recently been identified as part of the neuropsychological profile of AD, and impaired selective attention occurs early in AD (Levinoff et al., 2004).

Patients with insomnia also often present with impaired selective attention (Dai et al., 2021; Norell‐Clarke et al., 2014). A study exploring the correlates of FC of the attention network in insomnia patients found (Perrier et al., 2023) that insomnia patients exhibit stronger connectivity of the thalamic portion of the arousal circuit to the frontal and occipital gyri than good sleepers; the researchers suggested that insomnia patients may mobilize more cortical resources in visuomotor areas to direct attention. Further studies revealed (Krizan & Kerschensteiner, 2022) that the thalamus influences the transmission of visual information in the cortex and the onset of attention regulation by maintaining normal levels of cortical activity and neural oscillations. This may explain the enhanced FC between the occipital thalamus and superior occipital gyrus in insomnia patients with MCI; we speculate that the thalamocortical circuit is involved in the cognitive regulation of insomnia patients and that the abnormal FC between the thalamus and visual cortex involved in attention processing may play a role in the central pathological process of early cognitive impairment in insomnia patients.

However, what is most significant is that our findings differ from those of previous studies, as we observed a significant enhancement of FC between the occipital thalamus and the right superior occipital gyrus in PI–MCI patients compared to simple‐PI patients, and this alteration was negatively correlated with the MoCA total score. This phenomenon suggests that PI–MCI patients may be facing more severe cognitive impairments. Although the enhanced FC may represent adaptive changes in response to cognitive impairment in PI patients, this adaptation does not seem to have a positive impact on cognitive function and is unable to effectively compensate for cognitive impairment. Research indicates that certain neuroplasticity and functional compensation mechanisms in the brain ensure daily functions through functional reorganization in the face of mild damage (Gardini et al., 2015). With the further aggravation of cognitive damage, the compensation mechanism is gradually unable to be sustained and eventually progresses to the “decompensated effect” (Poirier et al., 2021). We hypothesized that insomnia may accelerate the process of decompensation, meaning that individuals with PI–MCI may demonstrate a more extensive pattern of FC enhancement in multiple brain regions, as observed in this study. However, due to the fact that PI patients “overdraw” the compensatory capacity of these brain regions earlier and, therefore, may fall into the abyss of “decompensation” faster when further cognitive function is damaged. Certainly, this concept needs further validation in larger sample sizes in the future.

In addition, FC changes in the thalamus and prefrontal lobes were observed in insomnia patients in the present study. Extensive changes in the FC of the occipital thalamus with the right superior frontal gyrus, left middle frontal gyrus, and right infraorbital frontal gyrus were demonstrated in PI–MCI patients. The prefrontal lobe is the management center for various cognitive functions in humans (Luo et al., 2021). Several studies have observed synchronous firing between the thalamus and prefrontal lobes, and this synchronous association was only present in the phase of memory decoding and storage during a task and was strongly correlated with working memory (Bolkan et al., 2017; Hsiao et al., 2020). Another study found (Hauer et al., 2019) that the thalamus plays a crucial role in coordinating slow‐wave activity between the prefrontal cortex and the hippocampus and suggested that slow‐wave sleep may underlie the associated situational memory consolidation. This implies that insomnia may impair situational memory consolidation and working memory as cognitive impairment continues to progress in patients with insomnia.

## 6 CONCLUSION

Taken together, our results support the hyperarousal theory of insomnia. More importantly, we found that PI patients with MCI present altered FC between the occipital thalamus and the superior occipital gyrus and that this abnormal FC is strongly correlated with the severity of cognitive decline. We suggest that enhanced FC between the thalamus, which is involved in attention processing, and the superior occipital gyrus (part of the visual cortex) may play a role in the pathological process of the early onset of cognitive impairment in insomnia patients. Moreover, due to the fact that PI patients “overdraw” the compensatory capacity of these brain regions earlier, the PI–MCI may fall into the abyss of “decompensation” faster and face more severe cognitive impairments. This study expands our understanding of the development of specific disorders associated with abnormal FC between brain regions and illustrates avenues to explore neuroimaging mechanisms in PI patients with MCI in the future.

## AUTHOR CONTRIBUTIONS

**Lin Zhang**: Writing‐original draft; formal analysis; data curation; visualization; investigation; conceptualization. **Linxin Bai**: Investigation; data curation. **Zhenxing Guo**: Investigation; data curation. **Jiahui Gao**: Investigation. **Jingsong Wu**: Supervision.**Jia Huang**: Supervision. **Zhizhen Liu**: Writing‐review and editing; methodology; supervision; conceptualization.

## CONFLICT OF INTEREST STATEMENT

The authors declare no conflicts of interest.

### PEER REVIEW

The peer review history for this article is available at https://publons.com/publon/10.1002/brb3.3411.

## Data Availability

The data that support the findings of this study are available on request from the corresponding author.
